# Pancreatic Cancer and Platelets Crosstalk: A Potential Biomarker and Target

**DOI:** 10.3389/fcell.2021.749689

**Published:** 2021-11-10

**Authors:** Shaoshan Mai, Iwona Inkielewicz-Stepniak

**Affiliations:** Department of Pharmaceutical Pathophysiology, Faculty of Pharmacy, Medical University of Gdańsk, Gdańsk, Poland

**Keywords:** pancreatic cancer, platelets, TCIPA, platelet-derived factors, platelet extracellular vesicles, angiogenesis, organoids, biomarker

## Abstract

Platelets have been recognized as key players in hemostasis, thrombosis, and cancer. Preclinical and clinical researches evidenced that tumorigenesis and metastasis can be promoted by platelets through a wide variety of crosstalk between cancer cells and platelets. Pancreatic cancer is a devastating disease with high morbidity and mortality worldwide. Although the relationship between pancreatic cancer and platelets in clinical diagnosis is described, the interplay between pancreatic cancer and platelets, the underlying pathological mechanism and pathways remain a matter of intensive study. This review summaries recent researches in connections between platelets and pancreatic cancer. The existing data showed different underlying mechanisms were involved in their complex crosstalk. Typically, pancreatic tumor accelerates platelet aggregation which forms thrombosis. Furthermore, extracellular vesicles released by platelets promote communication in a neoplastic microenvironment and illustrate how these interactions drive disease progression. We also discuss the advantages of novel model organoids in pancreatic cancer research. A more in-depth understanding of tumor and platelets crosstalk which is based on organoids and translational therapies may provide potential diagnostic and therapeutic strategies for pancreatic cancer progression.

## Introduction

Significant interaction of tumor cells with platelets is a longstanding concept. Preclinical and clinical studies showed that tumorigenesis, progression, angiogenesis, and metastasis can be promoted by platelets through a wide variety of crosstalk between platelets and cancer cells. Correlations between high platelet counts and poor prognosis are often described for lung, colon, breast, kidney, ovarian, and pancreatic cancers. Platelet-based biomarkers as liquid biopsy for cancer patients will be a potential platform for improving diagnosis. High risk of venous thrombosis and metastasis have close interrelations with platelets in patients with pancreatic cancer (Sylman et al., 2017). However, studies focused on crosstalk between pancreatic cancer and platelets in different ways are not fully explored compared with other tumors. For example, platelet α-granules contain many different bioactive factors such as vascular endothelial growth factor (VEGF), platelet-derived growth factor (PDGF), epidermal growth factor (EGF), endostatin, angiostatin, platelet factor-4 (PF4), or thrombospondin, the exact mechanisms of granule release and whether they can be selectively manipulated in pancreatic cancer requires more study. In addition, platelet derived-microparticles and RNA profile alteration are prospective directions for pancreatic cancer diagnosis and treatment (Thaler et al., 2012; Best et al., 2015). At the same time, multiple drugs have been developed to interfere with cancer growth or metastasis by inhibiting the functions of platelets. These drugs are in pre-clinical development or already in clinical treatment, which target platelet receptors, inhibit platelet granule release, or interfere with platelet-related enzymes (Dovizio et al., 2013; Elwood et al., 2016; Sun et al., 2019). In this review, we summarize recent discoveries in the field of pancreatic cancer and platelets. Compared with other cancer research with platelets, we discuss the potential exploration in pancreatic cancer. Moreover, we propose a suitable research model for pancreatic cancer and platelets investigation, which provides an insight for further study.

## Overview of Pancreatic Cancer

Pancreatic cancer remains one of the most lethal neoplasms worldwide. There are three histological classifications of pancreatic cancer. The pancreatic ductal adenocarcinoma (PDAC) comes from the duct cells of exocrine tissue, which represents the most common (> 90%) type. There is one subtype that exhibits both characteristics of adenocarcinoma and squamous carcinoma. It is adenosquamous carcinomas concerning 1–4% of the exocrine malignancies. The other types are acinar and neuroendocrine tumors. According to Globocan estimates, there were more than 495,773 new patients diagnosed and about 466,000 deaths of pancreatic cancer in 2020.

From precursor to invasive cancer, there are three well-defined PDAC precursor lesions: Intraductal papillary mucinous neoplasms (IPMNs), mucinous cystic neoplasms (MCNs), and pancreatic intraepithelial neoplasm (PanIN), the latter being the most frequent precursor lesion which further divided into PanIN-1, 2, and 3 (Hezel et al., 2006; Hruban et al., 2007).

Pancreatic cancer often does not cause symptoms in the early stages, which makes it difficult to diagnose. Common symptoms include tummy pain or back pain, weight loss, indigestion, losing appetite, diarrhea, constipation, jaundice, blood clots, and fatigue. These symptoms can have many causes, and are unlikely to be pancreatic cancer. Thus, different kinds of tests are essential for diagnosis. The tests used to diagnose pancreatic cancer include 1. Blood test, such as blood cell count, liver and kidney function, or tumor markers (such as CA19-9, CEA, B72.3); 2. Ultrasound scan of the abdomen. 3. Computerized tomography (CT) scan or Positron emission tomography (PET-CT) scan. 4. Magnetic resonance imaging (MRI) scan or Magnetic resonance cholangio-pancreatography (MRCP). 5. Endoscopic ultrasound scan (EUS) or with/without Biopsy. 6. Endoscopic retrograde cholangio-pancreatography (ERCP) is usually used if the bile duct is blocked. 7. Laparoscopy. According to these tests, the information about tumor size, burden, and local vessel involvement can be achieved that necessary to determine the TNM (Tumor, Nodes, Metastases) stage (van Roessel et al., 2018).

Currently, the treatments for pancreatic cancer are chemotherapy, surgery, radiation therapy, targeted therapy, and immunotherapy. Only about 20% of people diagnosed with pancreatic cancer are eligible for surgical treatment because most are found after the disease has already spread. The kinds of surgery that are performed depend on the purpose of the surgery. For example, the Whipple procedure, which is carried on if the tumor is located only in the head of the pancreas. However, a Distal pancreatectomy is commonly done if the cancer is in the tail of the pancreas. Usually, surgery will combine with systemic therapy or/and radiation therapy. Adjuvant therapy is given after surgery. Sometimes, a few treatments are used to shrink a tumor before surgery, this is called neoadjuvant therapy or pre-operative therapy. Radiation therapy is performed by high-energy x-rays or other particles to destroy cancer cells such as traditional radiation therapy, stereotactic body radiation (SBRT) or cyberknife, proton beam therapy. Typically, chemotherapy will combine with radiation therapy at the same time, which is named radiosensitization. Chemotherapy is the main type of systemic therapy and includes an intravenous tube placed into a vein by a needle or orally. One type or a combination of different medications are used in patients. Currently, the drugs approved for pancreatic cancer are: Fluorouracil (5-FU), Capecitabin, Gemcitabine, Erlotinib, Leucovorin, Irinotecan, Nab-paclitaxel, Nanoliposomal irinotecan, and Oxaliplatin. In addition, there are some targeted treatments that focus on the specific genes of the cancer, proteins, or the tissue environment. For example, Erlotinib blocks epidermal growth factor receptor (EGFR), Olaparib influences a hereditary *BRCA* mutation, and Larotrectinib can be used for *NTRK* fusion (Wang et al., 2015; Filippi et al., 2021; Tutt et al., 2021). Moreover, Immunotherapy has become popular in recent years. Immune checkpoint inhibitors are an option for treating pancreatic cancer with high microsatellite instability (MSI-H), which include anti-PD-1 antibodies such as pembrolizumab (Diaz et al., 2017). Different treatment options are dependent on the stage of the tumor. For instance, resected patients’ chemotherapy will be gemcitabine or 5-FU based treatment, however, metastatic cancer will use Gemcitabine plus Nab-paclitaxel or a combination of 5-FU, Leucovorin, Irinotecan, and Oxaliplatin called FOLFIRINOX. Nevertheless, surgery is not the main method of treatment. The appropriate therapeutics are referenced to European Society for Medical Oncology (ESMO) guideline (Ducreux et al., 2015).

## Platelet Function

Platelets derive from megakaryocytes, which exist in circulation for 5–7 days. The size is approximately 2–4 μm and their volume is about 7 μm^3^. A normal number of platelets ranges between 150,000 and 450,000 per microliter of blood. They are removed from blood vessels by macrophages and neutrophils and, leave the body by the spleen.

The primary role of platelets is to maintain hemostasis, by the formation of a “platelet clot” (Tomaiuolo et al., 2017). Following vascular damage, initial platelet tethering is mediated by the interaction between the GPIbα in the platelet receptor GPIb-IX-V and A1 domain of Von Willebrand factor (vWF) deposited in the subendothelial matrix of the injured vessel wall. After platelet tethering, GPVI and αIIβ1 receptors promote platelet adhesion and activation (Ruggeri and Jackson, 2013; Welsh et al., 2014). GPVI has a low affinity for collagen. The αIIβ1 maintains stable adhesion to collagen and reinforces GPVI-collagen interaction. Subsequent stable adhesion occurs *via* binding of fibronectin, αIIbβ3, laminin, and vWF. In addition, platelet adhesion will form positive feedback to initiate circulating platelets activation. The final step is platelet aggregation by the binding of fibrinogen or vWF to αIIbβ3.

Apart from hemostasis and thrombosis, platelets also play an important role in immune activities. Platelets are able to recognize and interact with microbial pathogens including bacteria, viruses, and parasites. Platelet bounding shifts fee *L. monocytogenes* from “fast” clearance into CRIg-dependent “slow” clearance pathways (Broadley et al., 2016). In addition, different platelet receptors have various effects on cancer progression (Supplementary Table 1). Platelets express toll-like receptors from TLR1 to TLR9 which identify molecular motifs called pathogen-associated molecular patterns (PAMPs) (Cognasse et al., 2015). Interaction between platelets and leukocytes, monocytes, and granulocytes are evidenced, which through different receptor-ligands such as P-Selectin, PSGL-1. Platelets are involved in angiogenesis. Their activation facilitates release, eliciting potent angiogenic responses. Moreover, the release of platelet-derived phospholipids and microparticles are as synergistic regulators of angiogenesis (Walsh et al., 2015). However, tumor growth can be aggravated by uncontrolled angiogenesis. Luminal breast cancer cells secret cytokines absorbed by platelets, which help vessel formation (Kuznetsov et al., 2012). Recently, it has been established that platelets play a crucial role in cancer cell metastasis. Platelets make contact with tumors by direct surface interactions, such as surface receptors and glycoproteins, indirect platelet growth factors, such as VEDF, TGF-β, and microparticles (MP) (Goubran et al., 2014). Platelet-derived signals, for example, CXCL5 and CXCL7 are required for the rapid recruitment of granulocytes to tumor cells to form early metastatic niches (Labelle et al., 2014). Colorectal cancer cell interaction with platelets produces chimeric extracellular vesicles like three types of microparticles that promote metastasis through EMT and endothelial activation (Plantureux et al., 2020). Activated platelets P-selectin interact with circulating tumor cell P-selectin ligands, which form aggregation and prevent shear force-induced tumor membrane damage (Coupland and Parish, 2014; Egan et al., 2014). Accordingly, platelets and tumor cell interaction promote tumor cells extravasation (Haemmerle et al., 2018; Marcolino et al., 2020).

## Crosstalk Between Pancreatic Cancer and Platelets

### Pancreatic Cancer Influences Platelets

#### Tumor Cell-Induced Platelet Aggregation and Thrombopoiesis

Tumor cell-induced platelet aggregation (TCIPA) is not a new concept, which can be traced back to the late nineteenth century. For pancreatic cancer cells, it was evidenced by six human cell lines, which can induce platelet aggregation *via* activation of thrombin (Heinmöller et al., 1995). Several molecular pathways are involved in pancreatic TCIPA. Activated phospholipase A2 enzymes release arachidonic acid (AA) which is a precursor of thromboxane A2 (TXA2). Cyclooxygenase 1 (COX-1) catalyzes the transformation of AA into TAX2, which is important for platelet aggregation. TAX2 can activate the thromboxane receptor-induced changes of platelet shape, activation of integrins, and degranulation. Moreover, the expression of COX-2 is reported as increased in tissues of pancreatic cancer (Ding et al., 2003; Sangkuhl et al., 2011). P-selectin and tissue factor (TF) accumulation are associated with platelet activation and platelet-rich thrombus (Wang et al., 2012; Mezouar et al., 2015; Hisada et al., 2017). The increased von Willebrand Factor (VWF), a large polymeric glycoprotein, is involved in the adhesion and aggregation of platelets. Cancer patients are associated with a higher risk of venous thrombosis. Especially, high VWF levels were observed in pancreatic, lung, brain, stomach, and colorectal cancer patients (Obermeier et al., 2019). Podoplanin on the surface of cancer cells induces platelet aggregation. Pancreatic cancer-associated fibroblasts (CAF) reportedly express podoplanin (Takemoto et al., 2017; Suzuki-Inoue, 2019). In addition, a recent study showed pancreatic cancer (Paca) cells can stimulate the rapid release of neutrophil extracellular traps (NETs) and promote thrombus formation (Abdol Razak et al., 2017).

#### Changes of Phenotype

In patients diagnosed with pancreatic cancer at the head of the pancreas, platelet count and concentration of vascular endothelial growth factor (VEGF) released from platelets were significantly increased (Sabrkhany et al., 2017). Mean platelet volume (MPV) is changed in different stages of pancreatic cancer (PC). MPV was elevated in PC patients with synchronous liver metastases and stage III-IV (Yin et al., 2018). However, MPV was decreased in resectable PC patients with poor prognoses (Yagyu et al., 2021). Best et al. (2015) evidenced mRNA profiles of tumor-educated blood platelets (TEPs) were different between *KRAS* mutant pancreatic cancer and *KARS* wild-type. TEPs RSL24D1 mRNA was negatively related to early pancreatic cancer compared to healthy controls (Xue et al., 2018). Moreover, the platelet proteome of patients with head of pancreas cancer (stage I-II) is significantly different from that of healthy individuals of equivalent sex and age (Sabrkhany et al., 2017, 2018).

#### Extracellular Vesicles Release and Alteration

Extracellular vesicles (EVs) are a means that facilitate the exchange of a broad array of molecules between adjacent or distant cells. Platelet EV cargo includes lipids, protein, nucleic acids, and organelles, which can enter lymph, bone marrow, and synovial fluid. EVs are classified into exosomes (30–150 nm), microvesicles—also referred as microparticles or ectosomes—(100–1,000 nm), and apoptotic bodies (1,000–3000 nm) (Ferreira et al., 2020). Platelet-derived extracellular vesicles (PDEVs) are the most abundant type of EVs in the circulation. Microparticle (MP) refers to particles released from the surface of cells, especially in the field of platelet research (Supplementary Table 2). Patients with pancreatic cancer had significantly increased levels of MP-associated TF activity compare with healthy controls (Tesselaar et al., 2007; Tilley et al., 2008). MP-TF activity had a strong association with mortality in pancreatic cancer, which could be a marker for aggressive cancer phenotype (Thaler et al., 2012). In addition, circulating microvesicles (MVs)-associated thrombin generation is different between patients and healthy control (Hellum et al., 2017).

#### RNA Profiles Alteration

Platelet messenger RNA (mRNA) profile is currently emerging as a new potential biomarker in cancer diagnosis. A study demonstrated platelets isolated from glioma or prostate cancer patients contained the cancer-associated mRNA transfer EGFRvIII and PCA3 (Nilsson et al., 2011). Recent studies highlighted pancreatic tumors can alter platelet RNA profiles (Best et al., 2015). Platelets from cancer patients contained tumor-associated RNA biomarkers, indeed, mRNA sequencing of tumor-educated platelets can identify pancreatic cancer patients with 96% accuracy.

### Platelets Are Involved in Pancreatic Tumor Progression

#### Platelets and Platelet-Derived Factors Are Biomarkers for Diagnosis and Prodiagnosis

MPV is associated with the overall survival of pancreatic cancer patients (Yin et al., 2018). Particularly, large platelet size is associated with poor outcomes in patients with metastatic pancreatic cancer (Lembeck et al., 2019). However, reduced MPV levels predict shorter survival in patients after surgery (Yin et al., 2020). There is a novel scoring system based on hemostatic parameters that showed platelet count was an independent prognostic factor in advanced pancreatic cancer (Zhang et al., 2019). In many studies, CA19-9 decrease during treatment has been related to longer survival of pancreatic cancer. Moreover, the correlation of CA19-9 decreases; overall survival was stronger in advanced pancreatic cancer with fewer platelets (Chen et al., 2019). The preoperative platelet to lymphocyte ratio (PLR) was reported to be a significant independent prognostic factor in patients (Song et al., 2017). In addition, the increased PLR is also related to a poor long-term prognosis in resected pancreatic cancer (Yu et al., 2018; Negoi et al., 2019). Combining PLR and CA19-9 values could allow earlier diagnosis of pancreatic cancer patients with type 2 diabetic patients (Qin et al., 2019). Preoperative platelet-to-albumin ratio (PAR) was reported as a novel significant independent prognostic index for disease-free survival (DFS) and overall survival (OS) in patients after pancreatic resection (Shirai et al., 2017). Moreover, platelet receptors and platelet-derived factors are also involved in cancer progression (Supplementary Table 1). The platelet-derived growth factor (PDGF) signaling pathway plays an important role in the progression of pancreatic cancer. Duan et al. (2018) analyzed three published genome-wide association study datasets to observe genetic variants in the PDGF subunit B gene associated with pancreatic cancer risk in European populations. High levels of circulating PDGF-AA serve as a predictor of poor cancer-specific survival, whereas high levels of PDGF-BB are associated with a favorable prognosis (Rahbari et al., 2011; Kahlert et al., 2014). In addition, PDGFR beta (PDGFRβ) is more frequently expressed in primary endocrine pancreatic tumors (EPTs) and metastases as compared to normal endocrine pancreatic tissue (Fjällskog et al., 2007). PDGFRβ is a marker of activated pancreatic stellate cells (PSCs) which play a vital role in desmoplasia. Higher expression of PDGFRβ matched shorter prognosis as well as lymphatic invasion and lymph node metastasis (Yuzawa et al., 2012).

#### Platelets Accelerate Proliferation and Angiogenesis

Recent researches evidence that platelets have a direct effect on tumor cell proliferation. PANC-1 cancer cell proliferation was potentiated by human platelets in a manner dependent on the upregulation and activation of the oncoprotein c-MYC (Mitrugno et al., 2017). Platelet-derived growth factor (PDGF) is an important cytokine in pro-proliferative and invasion signaling, which plays a key role in the regulation of interactions between pancreatic cancer cells and adjacent stroma (Haqq et al., 2016). Moreover, dual-specificity phosphatase 28 (DUSP28) regulates chemo-resistance and migration in pancreatic cancers. PDGF-AA was evidenced in a public microarray database and *in vitro* assay, which is a critical role in pancreatic cancer malignancy. In addition, DUSP28 and PDGF-AA formed an acquired autonomous autocrine-signaling pathway. Targeting DUSP28 inhibited the tumor growth and migratory features through the blockade of PDGF-AA expression and intracellular signaling (Lee et al., 2017). Mucin 1 (MUC1), a transmembrane mucin glycoprotein, regulates PDGFA expression and secretion in pancreatic cancer cells, accordingly, influences the proliferation of pancreatic tumors (Sahraei et al., 2012). There are some treatments for targeting PDGF to decrease the proliferation of pancreatic cancer cells (Inoue et al., 2017; Salem et al., 2020). PDGF-BB mediates pancreatic cancer growth via regulation of the Hippo/Yes-associated protein signaling pathway (Li et al., 2021). NF-κB is the downstream stage of the AA pathway. NF-κB and activator protein 1(AP-1) can bind to COX-2, lipoxygenases (LOXs), and phospholipase A2 (PLA2), which play a pro-tumorigenic role in pancreatic cancer (Gong et al., 2014; Matejcic et al., 2018). COX-2 is only expressed in pancreatic islets and has no expression in normal exocrine pancreatic tissues. Numerous clinical studies reported that mRNA and protein expression of COX-2 are up-regulated in pancreatic cancer. The use of aspirin, the major pharmacological inhibitor of COXs, was negatively related to the incidence risk of pancreatic cancer (Sun et al., 2019). Moreover, low-dose aspirin (81–162 mg orally daily) is relatively selective for COX-1 inhibition, interaction with circling tumor cells, and platelet aggregation. However, two large cohort studies reported regular aspirin or non-aspirin NSAID use was not associated with future risk of pancreatic cancer. There is only a possible reduction in patients of pancreatic cancer with diabetes (Khalaf et al., 2018). Pancreatic cancer cells express the purinergic receptor P2Y12, that is an ADP receptor found mainly on platelets. Ticagrelor, a P2Y12 inhibitor, decreases the survival signals initiated in cancer cells by platelet-derived ADP and ATP (Elaskalani et al., 2017). Arachidonate 12-lipoxygenase (ALOX12) and 12-hydroxyeicosatetraenoic acid contribute to stromal aging-induced progression of pancreatic cancer (Sarsour et al., 2020). 5-lipoxygenases (5-LOX) were found overexpressed in the tissues of pancreatic cancer, the inhibition of 5-LOX induces apoptosis in pancreatic cancer cells (Zhou et al., 2015). In addition, P-selectin deficiency and soluble P-selectin abolish platelet deposition within tumors, decreasing the secretion of vascular endothelial growth factor and angiogenesis, thereby suppressing tumor growth (Qi et al., 2015). Rivipansel inhibits selectins and decreases the recruitment of plasma cells in multiple myeloma (Azab et al., 2012). Crizanlizumab, a selective blocking antibody of P-selectin, also is indicated as a potential treatment option for patients with pancreatic cancer in the future. Selected clinical trials of glycobiology-targeted therapeutics for pancreatic cancer are in Phase I, for example MVT-5873 and MVT-10775 (Smith and Bertozzi, 2021). The first study for platelets involved in tumor angiogenesis is by Pinedo et al. (1998) They proposed platelets as a rich source of stimulators and inhibitors of angiogenesis and their interaction with the endothelium. Starlinger et al. (2011) compared different methods to get circulating platelet-stored angiogenesis factors in pancreatic cancer patients, and a significant increase of thrombospondin 1(TSP-1) and platelet factor 4 (PF-4) were determined. The overexpression of VEGF and platelet-derived endothelial cell growth factor (PD-ECGF) protein significantly correlated with high microvessel density (MVD) in patient tissues of pancreatic cancer (Fujimoto et al., 1998). VEGF is a chemotactic vascular permeability factor stored in α-granules and released from activated platelets. The study evidenced that VEGF expression correlated significantly with increased intratumoral microvessel density (IMD), which are important regulators of pancreatic tumor angiogenesis and predictive of benefit from adjuvant therapy (Khorana et al., 2005). Platelet factor 4 (PF-4) inhibits angiogenesis *in vivo* and *in vitro* (Maione et al., 1990). PF-4 modulates fibroblast grow factor 2 (FGF-2) activity (Perollet et al., 1998). However, a study reported that FGF-1 and FGF-2 treatment led to the induction of phosphorylation of E-cadherin and β-catenin on tyrosine residues, resulting in angiogenesis in pancreatic cancer cells (El-Hariry et al., 2001). Accordingly, FGFR also plays a key role in tumor angiogenesis, downregulations of FGFR-2 led to decreased phosphorylation of ERK and VEGF-A in PDAC cells after FGF-2 stimulation (Compagni et al., 2000; Kang et al., 2019). Platelets are the sole source of EGF in circulation, however, inhibition of EGFR tyrosine kinase activity suppresses pancreatic tumor angiogenesis (Bruns et al., 2000). Interleukin-8 (IL-8) is a chemokine related to PF-4, which activates G-protein coupled receptors as a pro-angiogenic factor (Le et al., 2000). Platelets contain 40–100 times more TGF-β than other non-neoplastic cells (Assoian et al., 1983). Although TGF-β is a pro-angiogenic factor in many cancers, the role in PDAC is controversial. TGF-β interferes with a soluble TβRII, which suppresses pancreatic cancer angiogenesis (Rowland-Goldsmith et al., 2002). Contrarily, other studies evidenced TGFβ1 is overexpressed in PDAC, and it induces plasminogen activator inhibitor-1(PAI-1) expression in pancreatic cancer cells, promoting angiogenesis *in vivo* (Andreasen et al., 2000). TGF-β can promote stromal activation, which induces angiogenesis and attenuates a productive anti-tumor immune reaction (Hinz et al., 2007; Kano et al., 2007). Similarly, because pancreatic cancer has a highly hypoxic microenvironment, in which hypoxia-inducible factor-1α (HIF-1α) is activated. HIF-dependent pathways subsequently activate HGF/c-MET signaling pathway in pancreatic tumor cells, and induce angiogenesis (Kitajima et al., 2008). HDF can also be anti-angiogenic due to alternative splicing of the α-chain (Date et al., 1998). Platelets can induce several matrix metalloproteinase (MMPs) in platelet-tumor cell interactions. Integrin engagement leads to the secretion of MMP-2, MMP-9 and surface expression of MT1-MMP. MMP-9 is essential to angiogenesis in pancreatic cancer mice model (Nakamura et al., 2007). Expression of MMP-2 and MMP-9 mRNA are associated with microvessel density in pancreatic cancer patients (Xiang et al., 2017). Platelet-derived microparticles (PMP) can enhance tumor growth by the release of potent growth factors in the tumor micro-environment (Goubran et al., 2015).

#### Platelets Facilitate Metastasis

That platelets promote tumor metastasis is not a new idea. Activation of platelets and the TF-thrombin-PAR-1 pathway are reported to promote metastasis of PDAC cells (Yang et al., 2019). The activation of platelets increases lysophosphatidic acid (LPA) release from platelets, LPA in turn enhanced tumor cell invasiveness and cell migration (Yoshikawa et al., 2013). In addition, vWF-platelet interactions also promote a number of metastases (Patmore et al., 2020). Tumor cell-derived MMPs can elicit platelet activation, accordingly, platelet-activating factors can induce an increased MMP expression, and inhibition of MMP reduces both growth of pancreatic cancer metastases and the death rate (Jimenez et al., 2000). High TGF-β induced expression in PDAC patients is associated with pancreatic cancer cell migration (Costanza et al., 2019). Moreover, TGF-β has a role as a suppressor of stromal promotion or through alterations in pancreatic stellate cell MMP profiles with subsequent inhibition of pancreatic cancer cell migration (Tjomsland et al., 2016). Platelets secrete a mountain of growth factors and chemokines, such as VEGF, PDGF, CXCL5, to help establish metastatic foci, neovessel formation, and metastasis. In addition, inhibition of platelets activation prevents the P-selectin and integrin-dependent accumulation of pancreatic cancer cell microparticles and reduces metastasis (Mezouar et al., 2015). ADAM9 contributes to vascular invasion and is involved in metastasis (Oria et al., 2019). Epithelial-mesenchymal transition (EMT), a transient and reversible process, promotes cell motility, invasion, and dissemination of cancer cells out of the tumor microenvironment. Extravasated platelet aggregation is associated with the first step in the formation of EMT. Primary tumor cells surrounded by platelets exhibited characteristics of EMT in pancreatic cancer (Miyashita et al., 2015).

#### Platelets Improve Pancreatic Cancer Chemotherapy Resistance

A few studies have shown platelets can influence the efficacy of chemotherapy. Platelets increase the resistance of colon and ovarian cancer to 5-fluorouracil and paclitaxel (Radziwon-Balicka et al., 2012). Platelet factors hinder the cytotoxicity of sorafenib and regorafenib in hepatocellular carcinoma by increasing the phosphorylation of ERK and p38 (D’Alessandro et al., 2014). Elaskalani et al. (2017) evidenced that platelet-derived ADP and ATP promote pancreatic cancer cell survival and gemcitabine resistance. Ticagrelor, an inhibitor of ADP-P2Y12 axis, enhances chemotherapeutic efficacy in pancreatic cancer cells by targeting the Novel P2Y12-AKT pathway (Elaskalani et al., 2020). In addition, platelets also drive EMT to promote chemotherapy resistance. β1 integrin-dependent interaction within the tumor microenvironment may alter tumor response to chemotherapy.

## Conclusion and Potential Direction

**FIGURE 1:**
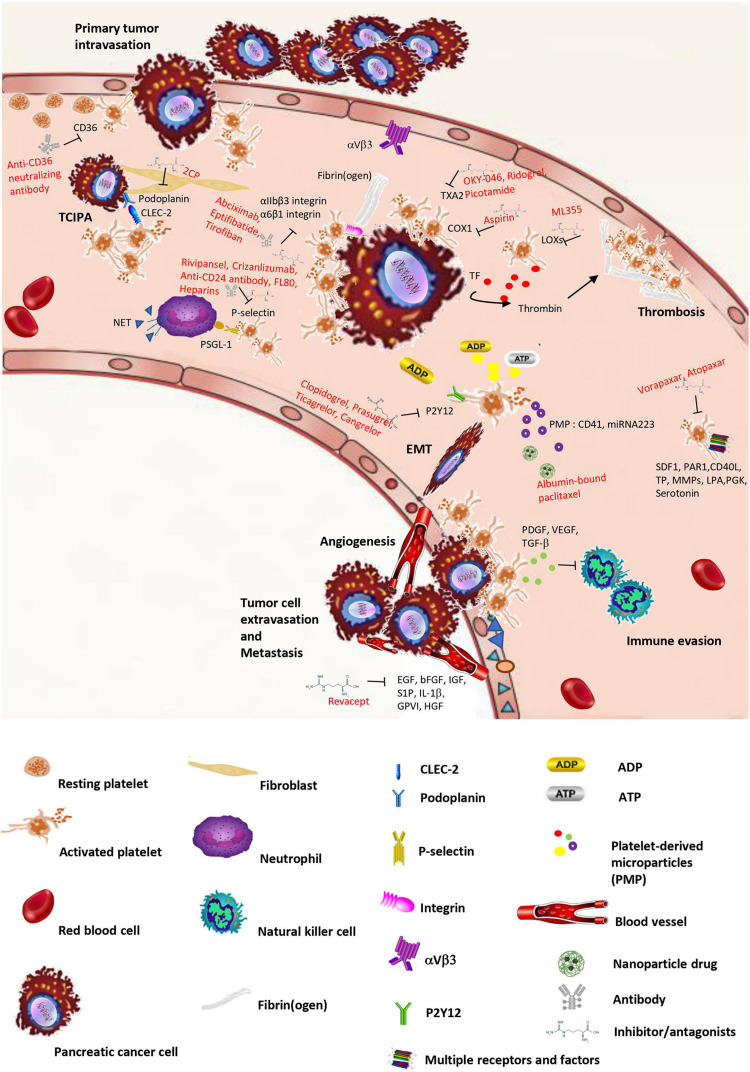
Schematic overview of crosstalk between platelets and pancreatic cancer. Pancreatic cancer can stimulate platelet activation and aggregation (TCIPA), change platelet shapes, and increase platelets’ release of microparticles with loading factors. However, platelet activation releases different kinds of factors, small molecules, and microparticles, which facilitate tumor growth, invasion, and metastasis. In the bloodstream, the tumor cell is surrounded by platelets that protect the encircled tumor cell from immune attack. In addition, platelets increase the properties of cancer cell adhesion and extravasation, thereby supporting cancer cell transmigration and metastasis. The intervention or inhibition of potential drugs in crosstalk between cancer and platelets are shown in the process.

This review highlights evidence for crosstalk between pancreatic cancer and platelets. Cancer influences platelets in different ways, such as TCIPA, thrombopoiesis, phenotype, and extracellular vesicles release. However, platelets are not only bystander cells in circulation, but also play a vital role in primary tumor growth and in the whole metastatic process (Figure 1). Although these relationships between platelets and cancer are reported in various kinds of tumors, they are still not full evidenced in pancreatic cancer. Platelet-related factors, chemokines, signaling pathways, and even microparticles are less explored in pancreatic cancer compared with other neoplasms (Supplementary Tables 1, 2).

Pancreatic cancer can manifest as exocrine or endocrine tumors, depending on the cell of origin. PDAC represents 90% of pancreatic malignancies. Patients are often diagnosed in the advanced staged, leading to an overall 5-year survival rate below 10%. To find a suitable model is necessary for pancreatic cancer fundamental research and clinical application. There are different *in vitro* and *in vivo* models to study the process of pancreatic cancer (Supplementary Table 3). Compared with the advantages and disadvantages of these models, three-dimensional organoid culture will be an optimal choice to study pancreatic cancer progression. Organoids can maintain cell polarity, closely resemble molecular features, and interact with an extracellular matrix (Boj et al., 2015). Platelets as biomarkers are to be a platform for detecting pancreatic cancer (Sabrkhany et al., 2021). Cancer-associated platelets represent a liquid biopsy for diagnosis. In addition, platelets play an important role in thrombocytosis, cancer dissemination, immune surveillance, angiogenesis, recruitment of neutrophils/monocytes, and tumor cell extravasation. Establishing a co-culture model of platelets and pancreatic cancer is inevitable. Some research exists that develops co-culture pancreatic cancer organoids with immune cells, cancer-associated fibroblasts (CAF) (Tsai et al., 2018; Holokai et al., 2020), however, the 3D culture environment for platelets is still in a very early development stage. Rothan et al. (2014) found a 3D culture environment increases the efficacy of platelet-rich plasma release in prompting skin fibroblast differentiation and extracellular matrix formation. Because platelets were defined as one part of blood cells, a perspective on applications of human blood cell culture and organoids is born (Seghatchian and Amiral, 2020). In fact, xenograft modes involving the transplantation of pancreatic tumor organoids have been shown to generate the full spectrum of tumor progression (Miyabayashi et al., 2020). Nevertheless, the development of co-culture pancreatic cancer organoids with platelets can imitate crosstalk between pancreatic cancer, extracellular matrix and platelets in tumor microenvironment. Accordingly, the underlying mechanism of interplay between pancreatic cancer and platelets will be uncovered. Furthermore, clinical and preclinical use of antiplatelet therapies in cancer will be improved, for example, aspirin, dipyridamole, RA-23 (cAMP-PDE inhibitor), clopidogrel (Tzanakakis et al., 1993; Mezouar et al., 2015). Using platelets as a drug delivery system (DDS) was demonstrated by Lyde et al. (2015). Synthetic nanoparticles through coating with platelet membranes or platelet mimicry approach could be a smart DDS against cancer in the future (Burnouf et al., 2018). However, the double-sided effect of tumor microenvironment on platelets targeting nanoparticles is reported that the homing nanoparticles could realize the targeting ability, photo-thermal effect, and tumor immunotherapeutic ability in the accessible tumor but not the hypovascular tumor such as pancreatic cancer (Chen et al., 2018).

In summary, pancreatic cancer has effects on phenotype, aggregation, factors release, RNA profile of platelets, conversely, platelets influence pancreatic cancer progression. To fully reveal the crosstalk between platelets and pancreatic cancer is a prospective direction for clinical diagnosis and treatment.

## Author Contributions

Both authors listed have made a substantial, direct and intellectual contribution to the work, and approved it for publication.

## Conflict of Interest

The authors declare that the research was conducted in the absence of any commercial or financial relationships that could be construed as a potential conflict of interest.

## Publisher’s Note

All claims expressed in this article are solely those of the authors and do not necessarily represent those of their affiliated organizations, or those of the publisher, the editors and the reviewers. Any product that may be evaluated in this article, or claim that may be made by its manufacturer, is not guaranteed or endorsed by the publisher.
