# Supplementary material for: Pancreatic Cancer and Platelets Crosstalk: A Potential Biomarker and Target
Source: Front Cell Dev Biol. 2021 Nov 10;9:749689. doi: 10.3389/fcell.2021.749689 (PMC8631477; doi:10.3389/fcell.2021.749689)
Supplement: Supplementary file 1 [file Data_Sheet_1.docx]

Supplementary Material

| **Table 1. Summary of Platelets Receptors, Platelets Released Factors with Known Roles in Reported Tumors** | | | | | |
| --- | --- | --- | --- | --- | --- |
| **Molecule** | **Reported tumor types** | **Comments** | **Pancreatic cancer reported** | **Reference example** | |
| GPIbα | melanoma, liver cancer, ovarian cancer, lung cancer | metastasis, thrombopoietin generation | No | (Jain et al., 2007),(Malehmir et al., 2019) | |
| aIIbβ3 integrin | melanoma, breast cancer, ovarian cancer, prostate cancer, cervical cancer, glioblastoma | metastasis | Yes | (Felding-Habermann et al., 2001),(Bakewell et al., 2003) | |
| a6β1 integrin | breast cancer, colon cancer, prostate cancer, lung cancer, intrahepatic cholangiocarcinoma, ovarian cancer | metastasis | Yes | (Mammadova-Bach et al., 2016),(Sawai et al., 2003) | |
| P-selectin | colorectal cancer, prostate cancer, lung cancer, liver cancer, neuroblastoma, breast cancer, gastric cancer, melanoma | metastasis, tumor growth, microthrombi | Yes | (Becker et al., 2017),(Haschemi et al., 2021) | |
| TLR4 | melanoma, lung cancer | platelets activation, adhesion, metastasis | No | (Yu et al., 2014),(Ren et al., 2021) | |
| CLEC-2 | melanoma, brain cancer, lung cancer, head and neck cancer, ovarian cancer | prothrombotic promotion, metastasis, platelets aggregation | Yes | (Shirai et al., 2017),(Riedl et al., 2017) | |
| GPVI | melanoma, lung cancer, colon cancer, breast cancer, prostate cancer | metastasis | Yes | (Jain et al., 2009),(Haschemi et al., 2021) | |
| CD36 | oral squamous cell carcinoma, melanoma, breast cancer | platelets activation, metastasis | Yes | (Pascual et al., 2017),(Pang et al., 2019) | |
| LPA | breast cancer, colon cancer, prostate cancer | metastasis, cancer growth | Yes | (Boucharaba et al., 2004),(Liao et al., 2013) | |
| CD40L | melanoma, breast cancer, colon cancer, nasopharyngeal carcinoma, lung cancer, gastric cancer, ovarian tumors, rectal cancer | tumor growth, metastasis | Yes | (Chiodoni et al., 2006),(Angelou et al., 2018) | |
| NFE2 | melanoma, breast cancer | metastasis | No | (Camerer et al., 2004) | |
| PAR4 | melanoma, breast cancer, human chondrosarcoma, hepatocellular carcinoma, colon cancer, ovarian cancer | platelets activation, metastasis | No | (Camerer et al., 2004),(Zhang et al., 2018) | |
| Gαq | melanoma, lung cancer, ovarian cancer, lung cancer | metastasis, platelets activation | No | (Palumbo et al., 2005),(Cho et al., 2021) | |
| P2Y12 | lung cancer, melanoma, hepatocellular carcinoma, ovarian cancer | metastasis, tumor growth | Yes | (Wang et al., 2013),(Cho et al., 2017) | |
| EGF | oral squamous carcinoma, lung cancer, breast cancer, hepatocellular carcinoma | tumor growth, differentiation, metastasis, pro-angiogenesis | Yes | (Sierko and Wojtukiewicz, 2004),(Chen et al., 2018) | |
| PDGF | colorectal cancer, esophageal cancer, lung cancer, liver cancer, melanoma, breast cancer | pro-angiogenesis, proliferation, metastasis | Yes | (Olsen et al., 2019),(Li et al., 2021) | |
| VEGF | breast cancer, colorectal cancer, lung cancer, ovarian cancer, liver cancer, renal carcinoma | pro-angiogenesis, vascular integrity | Yes | (Jiang et al., 2017),(Karayiannakis et al., 2003) | |
| bFGF | renal carcinoma, lung cancer, ovarian cancer, melanoma, breast cancer | pro-angiogenesis, mitogenic, tumor progression | Yes | (Hoffmann et al., 2008),(Peterson et al., 2012) | |
| IGF | breast cancer, lung cancer, papillary thyroid cancer | Mitogenic, tumor progression, angiogenesis | Yes | (Pasanisi et al., 2008),(Trajkovic-Arsic et al., 2013) | |
| IL-1β | cervical cancer, colorectal cancer, lung cancer, hepatocellular carcinoma, myeloma | pro-angiogenesis | Yes | (Carmi et al., 2013),(Takagi et al., 2018) | |
| SDF-1 (CXCL12) | breast cancer, prostate cancer, renal carcinoma, lung cancer, ovarian cancer, skin squamous cell carcinomas | pro-angiogenesis, tumor progression, metastasis | Yes | (Bernat-Peguera et al., 2019),(Sleightholm et al., 2017) | |
| TP (PD-ECGF) | gastric cancer, endometrial carcinoma, lung cancer, colon cancer, gallbladder cancer, renal cancer, bladder cancer, cervical cancer, breast cancer | pro-angiogenesis, tumor progression, chemotherapy activation | Yes | (Fujimoto et al., 1998),(Furukawa et al., 2018) | |
| SERPINE1 (PAI-1) | gastric cancer, breast cancer, head and neck cancer, acute leukemia, hepatocellular carcinoma, rectal cancer | Pro-angiogenesis, tumor invasion | Yes | (Li et al., 2018),(Kubala and DeClerck, 2019) | |
| TGF-β | ovarian cancer, breast cancer, cervical cancer, colon cancer | tumorigenesis and progression, | Yes | (Guo et al., 2019),(Chen et al., 2013) | |
| MMPs  (MMP-1, -2, -3, -9, -14) | prostate cancer, colon cancer, breast cancer, fibrosarcoma | metastasis, tumor cell invasion | Yes | (Gresele et al., 2017),(Łukaszewicz-Zając et al., 2019) | |
| HGF | prostate cancer, hepatocellular carcinoma, rhabdomyosarcoma, squamous cell carcinoma, ovarian cancer | tumor progression, angiogenesis, metastasis | Yes | (Pothula et al., 2020),(Parizadeh et al., 2019) | |
| PF4 | lung cancer, liposarcoma, osteosarcoma, adenocarcinoma, colorectal cancer, breast cancer | inhibit tumor growth, anti-angiogenesis | Yes | (Cervi et al., 2008),(Pilatova et al., 2013) | |
| Angiopoietin 1 (Ang1) | prostate cancer, breast cancer, gastric cancer, colorectal cancer, | tumor progression, anti-angiogenesis, maintain vascular integrity | Yes | (Caine et al., 2004),(Caine et al., 2007) | |
| TSP1 | prostate cancer, lung cancer, colorectal cancer, ovarian cancer, breast cancer | enhance platelet aggregation, anti-angiogenesis | Yes | (Kerr et al., 2021),(Kasper et al., 2001) | |
| endostatin | lung cancer, colorectal cancer, melanoma, gastric cancer, nasopharyngeal carcinoma, hepatocarcinoma, breast cancer, esophageal cancer, biliary tumor, ovarian carcinoma, cervical cancer, osteosarcoma, soft-tissue sarcoma, glioma, NF2 schwannoma | anti-angiogenesis, metastasis | Yes | (Ohlund et al., 2008),(Di Vito et al., 2017) | |
| S1P | glioblastoma, breast cancer, colon cancer, thyroid follicular carcinoma, hepatocarcinoma | vascular permeability, invasion | Yes | (Visentin et al., 2006),(Nagahashi et al., 2014) | |
| TIMPs  (TIMP-1, -2, -4) | colorectal cancer, prostate cancer, gastric cancer, melanoma | anti-angiogenesis | No | (Wang et al., 2006),(Prokopchuk et al., 2018) | |
| PGK | hepatocellular carcinoma | tumor progression, | Yes | (Jiang et al., 2019),(Ho et al., 2010) | |
| ATP, ADP | ovarian cancer, breast cancer, prostate cancer, lung cancer | aggregation, platelet activation, adhesion | Yes | (Elaskalani et al., 2017),(Davis et al., 2020) | |
| histamine | lung cancer, medullary thyroid cancer, oral cancer colon cancer | proinflammatory, vascular permeability | No | (Massari et al., 2020),(Losurdo et al., 2018) | |
| serotonin | prostate cancer, bladder cancer, lung cancer, colorectal cancer, bile duct cancer, cholangiocarcinoma, breast cancer, hepatocellular cancer, glioma carcinoids | aggregation, angiogenesis, metastasis | Yes | (Sarrouilhe and Mesnil, 2019),(Xiao, 2020) | |
|  | | | | |  |

| **Table 2. Summary of Platelet-Derived Microparticles with Known Roles in Reported Tumors** | | | | |
| --- | --- | --- | --- | --- |
| **Platelet-Derived Microparticles** | **Reported tumor types** | **Comments** | **Pancreatic cancer reported** | **Reference example** |
| TF | myeloproliferative neoplasm, breast cancer, lung cancer, brain cancer, prostate, leukemia | active procoagulant properties | Yes | (Khorana et al., 2008),(Yenigürbüz et al., 2019) |
| CD61 | colorectal cancer, breast cancer | adhesion, aggregation | No | (Toth et al., 2008),(Stec et al., 2015) |
| CD41 | lung carcinoma, breast cancer, gastric cancer | adhesion, aggregation, metastasis | Yes | (Grande et al., 2019),(Yu et al., 2020) |
| P-selectin | breast cancer | binding to PSGL-1, activation, aggregation, adhesion | No | (Toth et al., 2008),(van der Zee et al., 2006) |
| CD63 | breast cancer, lung cancer | platelet activation, coagulation | No | (van der Zee et al., 2006),(Kanazawa et al., 2003) |
| VEGF | Prostate cancer | angiogenesis | No | (Helley et al., 2009) |
| bFGF | Prostate cancer | angiogenesis | No | (Helley et al., 2009) |
| PAR-1 | colon cancer | procoagulant activity | No | (Jia et al., 2015) |
| TGF-β | osteosarcoma, bladder, pleural mesothelioma | inflammation,  angiogenesis, | Yes | (Saito et al., 2018),(Chen et al., 2013) |
| miRNA-24 | colon cancer, lung cancer | mitochondrial depolarization,  induced apoptosis, inhibited tumor growth | No | (Michael et al., 2017) |
| miRNA-123 | breast cancer | migration, cell cycle | No | (Gasperi et al., 2019) |
| miRNA-223 | gastric cancer, lung cancer | proliferation, invasion, apoptosis | Yes | (Komatsu et al., 2015) |
| miRNA-233 | breast cancer | migration, cell cycle | No | (Gasperi et al., 2019) |
| miRNA-939 | ovarian cancer | invasion | No | (Tang et al., 2017) |

|  | Table 3. Comparison of PDAC preclinical models | | | | | |
| --- | --- | --- | --- | --- | --- | --- |
|  | **Type of model** | | **Short description** | **Advantages** | **Limitations** | **References** |
| In vitro | 2D cell line | PANC-1, HuP-T3, MIA PaCa-2, AsPC-1, BxPC-3, CFPAC-1, HuP-T4, PSN-1; CFPAC-1, BxPC-3, AsPC-1, PANC-1 and MIA PaCa-2 cell lines were used to screen the anticancer properties of drug conjugates to treat pancreatic cancer; Pancreatic cancer cell lines like HuP-T1, HuP-T3 and PANC-1 were used to suggest anti-sense therapy for the K-ras point mutation frequently observed in pancreatic cancers; Hup-T4 cell lines were used to investigate the role of the ARP2/3 complex in pancreatic cancer cell migration | | less time, easy handling and propagation, highly standardized, drug and biomarker rapid screening, less costs | lack of tumour heterogeneity, lack of tumour microenvironment, lack of stromal structure, genetic drift | (Heller et al., 2016),(Biau et al., 2016) |
|  | 3D organoids | human or mice tissue origin, primary cell culture | | moderate time, can be cultured and passage, cryopreserve, with stromal structure, similar tumour microenvironment, genetic diversity and tumour heterogeneity, personalized drug screening | technically sophisticated, moderate cost, need more detail characterization, require high quality patients tissues as sample | (Neal et al., 2018),(Boj et al., 2015) |
| In vivo | Genetically engineered mouse models | genetic analyses of tumour development and progression, spontaneous | | complete immune system, informative for involvement genetic mutation, stroma, metastasis, drug and biomarker discovery | cost high, time consuming, lack of tumour complexity, tumour develop instability | (Kong et al., 2020),(Westphalen and Olive, 2012) |
|  | chemically Induced animal models | Wistar and Lewis rats are injected intraperitoneally with azaserine to induce acinar cell carcinoma of the pancreas, with liver, lung and lymph node metastasis, hamsters induced | | identify known and emerging human risk factors and implement appropriate interventions | lack a typical duct-like structure and often occur alongside tumours of other organs (mammary, liver, kidney) | (Hayashi and Hasegawa, 1971),(Murphy et al., 2016),(Fujii et al., 1990) |
|  | cell line-derived xenografts | subcutaneous or in situ tumour formation, need cell line selection, tumorigenicity, nude mice as transplanted receiver | | moderate time, relatively inexpensive, drug safety and efficacy testing | limited stroma, genetic homogeneity, limited relation to tumour type, establishment rate is poor, cell lines lack biologic stability | (Logsdon et al., 2015),(Behrens et al., 2017) |
|  | patient tissue-derived xenografts | implant small tumours from a patient's pancreas into experimental immune compromised mice, simulating their native growth environment, drug screening, prediction of drug responses in preclinical “phase II” studies, biomarker validation | | inter-tumoral heterogeneity, conserved gene and phenotype, suitable for individualized therapy prediction, metastatic potential | time- and cost- consuming, Lack of immune components, lack of human tumour stroma, engraftment rate, limited to biopsy sample or resectable tumour sample, few replicates per tumour | (Jun et al., 2016) |
|  | patient organoids-derived xenografts | culture organoids firstly, orthotopically transplanted humanized mice, | | stromal involvement, assessment of malignant progression and metastasis, personalized therapy screening, cryopreserved, tumour replicable, tumour heterogeneity, restructure patient tumour microenvironment | high cost, time consuming, technically sophisticated, need biopsy or tissues | (Miyabayashi et al., 2020) |

**Reference:**

Angelou, A., Antoniou, E., Garmpis, N., Damaskos, C., Theocharis, S., and Margonis, G.A. (2018). The Role of Soluble CD40L Ligand in Human Carcinogenesis. *Anticancer Res* 38(5)**,** 3199-3201. doi: 10.21873/anticanres.12585.

Bakewell, S.J., Nestor, P., Prasad, S., Tomasson, M.H., Dowland, N., Mehrotra, M., et al. (2003). Platelet and osteoclast beta3 integrins are critical for bone metastasis. *Proc Natl Acad Sci U S A* 100(24)**,** 14205-14210. doi: 10.1073/pnas.2234372100.

Becker, K.A., Beckmann, N., Adams, C., Hessler, G., Kramer, M., Gulbins, E., et al. (2017). Melanoma cell metastasis via P-selectin-mediated activation of acid sphingomyelinase in platelets. *Clin Exp Metastasis* 34(1)**,** 25-35. doi: 10.1007/s10585-016-9826-6.

Behrens, D., Walther, W., and Fichtner, I. (2017). Pancreatic cancer models for translational research. *Pharmacol Ther* 173**,** 146-158. doi: 10.1016/j.pharmthera.2017.02.013.

Bernat-Peguera, A., Simón-Extremera, P., da Silva-Diz, V., López de Munain, M., Díaz-Gil, L., Penin, R.M., et al. (2019). PDGFR-induced autocrine SDF-1 signaling in cancer cells promotes metastasis in advanced skin carcinoma. *Oncogene* 38(25)**,** 5021-5037. doi: 10.1038/s41388-019-0773-y.

Biau, J., Chautard, E., Court, F., Pereira, B., Verrelle, P., Devun, F., et al. (2016). Global Conservation of Protein Status between Cell Lines and Xenografts. *Transl Oncol* 9(4)**,** 313-321. doi: 10.1016/j.tranon.2016.05.005.

Boj, S.F., Hwang, C.I., Baker, L.A., Chio, II, Engle, D.D., Corbo, V., et al. (2015). Organoid models of human and mouse ductal pancreatic cancer. *Cell* 160(1-2)**,** 324-338. doi: 10.1016/j.cell.2014.12.021.

Boucharaba, A., Serre, C.M., Grès, S., Saulnier-Blache, J.S., Bordet, J.C., Guglielmi, J., et al. (2004). Platelet-derived lysophosphatidic acid supports the progression of osteolytic bone metastases in breast cancer. *J Clin Invest* 114(12)**,** 1714-1725. doi: 10.1172/jci22123.

Caine, G.J., Lip, G.Y., and Blann, A.D. (2004). Platelet-derived VEGF, Flt-1, angiopoietin-1 and P-selectin in breast and prostate cancer: further evidence for a role of platelets in tumour angiogenesis. *Ann Med* 36(4)**,** 273-277. doi: 10.1080/07853890410026098.

Caine, G.J., Ryan, P., Lip, G.Y., and Blann, A.D. (2007). Significant decrease in angiopoietin-1 and angiopoietin-2 after radical prostatectomy in prostate cancer patients. *Cancer Lett* 251(2)**,** 296-301. doi: 10.1016/j.canlet.2006.11.026.

Camerer, E., Qazi, A.A., Duong, D.N., Cornelissen, I., Advincula, R., and Coughlin, S.R. (2004). Platelets, protease-activated receptors, and fibrinogen in hematogenous metastasis. *Blood* 104(2)**,** 397-401. doi: 10.1182/blood-2004-02-0434.

Carmi, Y., Dotan, S., Rider, P., Kaplanov, I., White, M.R., Baron, R., et al. (2013). The role of IL-1β in the early tumor cell-induced angiogenic response. *J Immunol* 190(7)**,** 3500-3509. doi: 10.4049/jimmunol.1202769.

Cervi, D., Yip, T.T., Bhattacharya, N., Podust, V.N., Peterson, J., Abou-Slaybi, A., et al. (2008). Platelet-associated PF-4 as a biomarker of early tumor growth. *Blood* 111(3)**,** 1201-1207. doi: 10.1182/blood-2007-04-084798.

Chen, H., Lan, X., Liu, M., Zhou, B., Wang, B., and Chen, P. (2013). Direct TGF-β1 signaling between activated platelets and pancreatic cancer cells primes cisplatin insensitivity. *Cell Biol Int* 37(5)**,** 478-484. doi: 10.1002/cbin.10067.

Chen, R., Jin, G., Li, W., and McIntyre, T.M. (2018). Epidermal Growth Factor (EGF) Autocrine Activation of Human Platelets Promotes EGF Receptor-Dependent Oral Squamous Cell Carcinoma Invasion, Migration, and Epithelial Mesenchymal Transition. *J Immunol* 201(7)**,** 2154-2164. doi: 10.4049/jimmunol.1800124.

Chiodoni, C., Iezzi, M., Guiducci, C., Sangaletti, S., Alessandrini, I., Ratti, C., et al. (2006). Triggering CD40 on endothelial cells contributes to tumor growth. *J Exp Med* 203(11)**,** 2441-2450. doi: 10.1084/jem.20060844.

Cho, M.S., Li, J., Gonzalez-Delgado, R., Lee, H., Vasquez, M., He, T., et al. (2021). The effect of platelet G proteins on platelet extravasation and tumor growth in the murine model of ovarian cancer. *Blood Adv* 5(7)**,** 1947-1951. doi: 10.1182/bloodadvances.2020003410.

Cho, M.S., Noh, K., Haemmerle, M., Li, D., Park, H., Hu, Q., et al. (2017). Role of ADP receptors on platelets in the growth of ovarian cancer. *Blood* 130(10)**,** 1235-1242. doi: 10.1182/blood-2017-02-769893.

Davis, P.J., Mousa, S.A., Schechter, G.P., and Lin, H.Y. (2020). Platelet ATP, Thyroid Hormone Receptor on Integrin αvβ3 and Cancer Metastasis. *Horm Cancer* 11(1)**,** 13-16. doi: 10.1007/s12672-019-00371-4.

Di Vito, C., Navone, S.E., Marfia, G., Abdel Hadi, L., Mancuso, M.E., Pecci, A., et al. (2017). Platelets from glioblastoma patients promote angiogenesis of tumor endothelial cells and exhibit increased VEGF content and release. *Platelets* 28(6)**,** 585-594. doi: 10.1080/09537104.2016.1247208.

Elaskalani, O., Falasca, M., Moran, N., Berndt, M.C., and Metharom, P. (2017). The Role of Platelet-Derived ADP and ATP in Promoting Pancreatic Cancer Cell Survival and Gemcitabine Resistance. *Cancers (Basel)* 9(10). doi: 10.3390/cancers9100142.

Felding-Habermann, B., O'Toole, T.E., Smith, J.W., Fransvea, E., Ruggeri, Z.M., Ginsberg, M.H., et al. (2001). Integrin activation controls metastasis in human breast cancer. *Proc Natl Acad Sci U S A* 98(4)**,** 1853-1858. doi: 10.1073/pnas.98.4.1853.

Fujii, H., Egami, H., Chaney, W., Pour, P., and Pelling, J. (1990). Pancreatic ductal adenocarcinomas induced in Syrian hamsters by N-nitrosobis(2-oxopropyl)amine contain a c-Ki-ras oncogene with a point-mutated codon 12. *Mol Carcinog* 3(5)**,** 296-301. doi: 10.1002/mc.2940030510.

Fujimoto, K., Hosotani, R., Wada, M., Lee, J.U., Koshiba, T., Miyamoto, Y., et al. (1998). Expression of two angiogenic factors, vascular endothelial growth factor and platelet-derived endothelial cell growth factor in human pancreatic cancer, and its relationship to angiogenesis. *Eur J Cancer* 34(9)**,** 1439-1447. doi: 10.1016/s0959-8049(98)00069-0.

Furukawa, T., Tabata, S., Yamamoto, M., Kawahara, K., Shinsato, Y., Minami, K., et al. (2018). Thymidine phosphorylase in cancer aggressiveness and chemoresistance. *Pharmacol Res* 132**,** 15-20. doi: 10.1016/j.phrs.2018.03.019.

Gasperi, V., Vangapandu, C., Savini, I., Ventimiglia, G., Adorno, G., and Catani, M.V. (2019). Polyunsaturated fatty acids modulate the delivery of platelet microvesicle-derived microRNAs into human breast cancer cell lines. *J Nutr Biochem* 74**,** 108242. doi: 10.1016/j.jnutbio.2019.108242.

Grande, R., Dovizio, M., Marcone, S., Szklanna, P.B., Bruno, A., Ebhardt, H.A., et al. (2019). Platelet-Derived Microparticles From Obese Individuals: Characterization of Number, Size, Proteomics, and Crosstalk With Cancer and Endothelial Cells. *Front Pharmacol* 10**,** 7. doi: 10.3389/fphar.2019.00007.

Gresele, P., Falcinelli, E., Sebastiano, M., and Momi, S. (2017). Matrix Metalloproteinases and Platelet Function. *Prog Mol Biol Transl Sci* 147**,** 133-165. doi: 10.1016/bs.pmbts.2017.01.002.

Guo, Y., Cui, W., Pei, Y., and Xu, D. (2019). Platelets promote invasion and induce epithelial to mesenchymal transition in ovarian cancer cells by TGF-β signaling pathway. *Gynecol Oncol* 153(3)**,** 639-650. doi: 10.1016/j.ygyno.2019.02.026.

Haschemi, R., Gockel, L.M., Bendas, G., and Schlesinger, M. (2021). A Combined Activity of Thrombin and P-Selectin Is Essential for Platelet Activation by Pancreatic Cancer Cells. *Int J Mol Sci* 22(7). doi: 10.3390/ijms22073323.

Hayashi, Y., and Hasegawa, T. (1971). Experimental pancreatic tumor in rats after intravenous injection of 4-hydroxyaminoquinoline 1-oxide. *Gan* 62(4)**,** 329-330.

Heller, A., Angelova, A.L., Bauer, S., Grekova, S.P., Aprahamian, M., Rommelaere, J., et al. (2016). Establishment and Characterization of a Novel Cell Line, ASAN-PaCa, Derived From Human Adenocarcinoma Arising in Intraductal Papillary Mucinous Neoplasm of the Pancreas. *Pancreas* 45(10)**,** 1452-1460. doi: 10.1097/mpa.0000000000000673.

Helley, D., Banu, E., Bouziane, A., Banu, A., Scotte, F., Fischer, A.M., et al. (2009). Platelet microparticles: a potential predictive factor of survival in hormone-refractory prostate cancer patients treated with docetaxel-based chemotherapy. *Eur Urol* 56(3)**,** 479-484. doi: 10.1016/j.eururo.2008.06.038.

Ho, M.Y., Tang, S.J., Ng, W.V., Yang, W., Leu, S.J., Lin, Y.C., et al. (2010). Nucleotide-binding domain of phosphoglycerate kinase 1 reduces tumor growth by suppressing COX-2 expression. *Cancer Sci* 101(11)**,** 2411-2416. doi: 10.1111/j.1349-7006.2010.01691.x.

Hoffmann, A.C., Mori, R., Vallbohmer, D., Brabender, J., Drebber, U., Baldus, S.E., et al. (2008). High expression of heparanase is significantly associated with dedifferentiation and lymph node metastasis in patients with pancreatic ductal adenocarcinomas and correlated to PDGFA and via HIF1a to HB-EGF and bFGF. *J Gastrointest Surg* 12(10)**,** 1674-1681; discussion 1681-1672. doi: 10.1007/s11605-008-0628-2.

Jain, S., Russell, S., and Ware, J. (2009). Platelet glycoprotein VI facilitates experimental lung metastasis in syngenic mouse models. *J Thromb Haemost* 7(10)**,** 1713-1717. doi: 10.1111/j.1538-7836.2009.03559.x.

Jain, S., Zuka, M., Liu, J., Russell, S., Dent, J., Guerrero, J.A., et al. (2007). Platelet glycoprotein Ib alpha supports experimental lung metastasis. *Proc Natl Acad Sci U S A* 104(21)**,** 9024-9028. doi: 10.1073/pnas.0700625104.

Jia, Y., Zhang, S., Miao, L., Wang, J., Jin, Z., Gu, B., et al. (2015). Activation of platelet protease-activated receptor-1 induces epithelial-mesenchymal transition and chemotaxis of colon cancer cell line SW620. *Oncol Rep* 33(6)**,** 2681-2688. doi: 10.3892/or.2015.3897.

Jiang, L., Luan, Y., Miao, X., Sun, C., Li, K., Huang, Z., et al. (2017). Platelet releasate promotes breast cancer growth and angiogenesis via VEGF-integrin cooperative signalling. *Br J Cancer* 117(5)**,** 695-703. doi: 10.1038/bjc.2017.214.

Jiang, Y., He, R., Jiang, Y., Liu, D., Tao, L., Yang, M., et al. (2019). Transcription factor NFAT5 contributes to the glycolytic phenotype rewiring and pancreatic cancer progression via transcription of PGK1. *Cell Death Dis* 10(12)**,** 948. doi: 10.1038/s41419-019-2072-5.

Jun, E., Jung, J., Jeong, S.Y., Choi, E.K., Kim, M.B., Lee, J.S., et al. (2016). Surgical and Oncological Factors Affecting the Successful Engraftment of Patient-derived Xenografts in Pancreatic Ductal Adenocarcinoma. *Anticancer Res* 36(2)**,** 517-521.

Kanazawa, S., Nomura, S., Kuwana, M., Muramatsu, M., Yamaguchi, K., and Fukuhara, S. (2003). Monocyte-derived microparticles may be a sign of vascular complication in patients with lung cancer. *Lung Cancer* 39(2)**,** 145-149. doi: 10.1016/s0169-5002(02)00441-5.

Karayiannakis, A.J., Bolanaki, H., Syrigos, K.N., Asimakopoulos, B., Polychronidis, A., Anagnostoulis, S., et al. (2003). Serum vascular endothelial growth factor levels in pancreatic cancer patients correlate with advanced and metastatic disease and poor prognosis. *Cancer Lett* 194(1)**,** 119-124. doi: 10.1016/s0304-3835(03)00047-8.

Kasper, H.U., Ebert, M., Malfertheiner, P., Roessner, A., Kirkpatrick, C.J., and Wolf, H.K. (2001). Expression of thrombospondin-1 in pancreatic carcinoma: correlation with microvessel density. *Virchows Arch* 438(2)**,** 116-120. doi: 10.1007/s004280000302.

Kerr, B.A., Harris, K.S., Shi, L., Willey, J.S., Soto-Pantoja, D.R., and Byzova, T.V. (2021). Platelet TSP-1 controls prostate cancer-induced osteoclast differentiation and bone marrow-derived cell mobilization through TGFβ-1. *Am J Clin Exp Urol* 9(1)**,** 18-31.

Khorana, A.A., Francis, C.W., Menzies, K.E., Wang, J.G., Hyrien, O., Hathcock, J., et al. (2008). Plasma tissue factor may be predictive of venous thromboembolism in pancreatic cancer. *J Thromb Haemost* 6(11)**,** 1983-1985. doi: 10.1111/j.1538-7836.2008.03156.x.

Komatsu, S., Ichikawa, D., Miyamae, M., Kawaguchi, T., Morimura, R., Hirajima, S., et al. (2015). Malignant potential in pancreatic neoplasm; new insights provided by circulating miR-223 in plasma. *Expert Opin Biol Ther* 15(6)**,** 773-785. doi: 10.1517/14712598.2015.1029914.

Kong, K., Guo, M., Liu, Y., and Zheng, J. (2020). Progress in Animal Models of Pancreatic Ductal Adenocarcinoma. *J Cancer* 11(6)**,** 1555-1567. doi: 10.7150/jca.37529.

Kubala, M.H., and DeClerck, Y.A. (2019). The plasminogen activator inhibitor-1 paradox in cancer: a mechanistic understanding. *Cancer Metastasis Rev* 38(3)**,** 483-492. doi: 10.1007/s10555-019-09806-4.

Li, S., Wei, X., He, J., Tian, X., Yuan, S., and Sun, L. (2018). Plasminogen activator inhibitor-1 in cancer research. *Biomed Pharmacother* 105**,** 83-94. doi: 10.1016/j.biopha.2018.05.119.

Li, T., Guo, T., Liu, H., Jiang, H., and Wang, Y. (2021). Platelet‑derived growth factor‑BB mediates pancreatic cancer malignancy via regulation of the Hippo/Yes‑associated protein signaling pathway. *Oncol Rep* 45(1)**,** 83-94. doi: 10.3892/or.2020.7859.

Liao, Y., Mu, G., Zhang, L., Zhou, W., Zhang, J., and Yu, H. (2013). Lysophosphatidic acid stimulates activation of focal adhesion kinase and paxillin and promotes cell motility, via LPA1-3, in human pancreatic cancer. *Dig Dis Sci* 58(12)**,** 3524-3533. doi: 10.1007/s10620-013-2878-4.

Logsdon, C.D., Arumugam, T., and Ramachandran, V. (2015). Animal Models of Gastrointestinal and Liver Diseases. The difficulty of animal modeling of pancreatic cancer for preclinical evaluation of therapeutics. *Am J Physiol Gastrointest Liver Physiol* 309(5)**,** G283-291. doi: 10.1152/ajpgi.00169.2015.

Losurdo, G., Principi, M., Girardi, B., Pricci, M., Barone, M., Ierardi, E., et al. (2018). Histamine and Histaminergic Receptors in Colorectal Cancer: From Basic Science to Evidence-based Medicine. *Anticancer Agents Med Chem* 18(1)**,** 15-20. doi: 10.2174/1871520616666160321115349.

Łukaszewicz-Zając, M., Gryko, M., Pączek, S., Szmitkowski, M., Kędra, B., and Mroczko, B. (2019). Matrix metalloproteinase 2 (MMP-2) and its tissue inhibitor 2 (TIMP-2) in pancreatic cancer (PC). *Oncotarget* 10(3)**,** 395-403. doi: 10.18632/oncotarget.26571.

Malehmir, M., Pfister, D., Gallage, S., Szydlowska, M., Inverso, D., Kotsiliti, E., et al. (2019). Platelet GPIbα is a mediator and potential interventional target for NASH and subsequent liver cancer. *Nat Med* 25(4)**,** 641-655. doi: 10.1038/s41591-019-0379-5.

Mammadova-Bach, E., Zigrino, P., Brucker, C., Bourdon, C., Freund, M., De Arcangelis, A., et al. (2016). Platelet integrin α6β1 controls lung metastasis through direct binding to cancer cell-derived ADAM9. *JCI Insight* 1(14)**,** e88245. doi: 10.1172/jci.insight.88245.

Massari, N.A., Nicoud, M.B., and Medina, V.A. (2020). Histamine receptors and cancer pharmacology: an update. *Br J Pharmacol* 177(3)**,** 516-538. doi: 10.1111/bph.14535.

Michael, J.V., Wurtzel, J.G.T., Mao, G.F., Rao, A.K., Kolpakov, M.A., Sabri, A., et al. (2017). Platelet microparticles infiltrating solid tumors transfer miRNAs that suppress tumor growth. *Blood* 130(5)**,** 567-580. doi: 10.1182/blood-2016-11-751099.

Miyabayashi, K., Baker, L.A., Deschênes, A., Traub, B., Caligiuri, G., Plenker, D., et al. (2020). Intraductal Transplantation Models of Human Pancreatic Ductal Adenocarcinoma Reveal Progressive Transition of Molecular Subtypes. *Cancer Discov* 10(10)**,** 1566-1589. doi: 10.1158/2159-8290.Cd-20-0133.

Murphy, B., Yin, H., Maris, J.M., Kolb, E.A., Gorlick, R., Reynolds, C.P., et al. (2016). Evaluation of Alternative In Vivo Drug Screening Methodology: A Single Mouse Analysis. *Cancer Res* 76(19)**,** 5798-5809. doi: 10.1158/0008-5472.Can-16-0122.

Nagahashi, M., Takabe, K., Terracina, K.P., Soma, D., Hirose, Y., Kobayashi, T., et al. (2014). Sphingosine-1-phosphate transporters as targets for cancer therapy. *Biomed Res Int* 2014**,** 651727. doi: 10.1155/2014/651727.

Neal, J.T., Li, X., Zhu, J., Giangarra, V., Grzeskowiak, C.L., Ju, J., et al. (2018). Organoid Modeling of the Tumor Immune Microenvironment. *Cell* 175(7)**,** 1972-1988.e1916. doi: 10.1016/j.cell.2018.11.021.

Ohlund, D., Ardnor, B., Oman, M., Naredi, P., and Sund, M. (2008). Expression pattern and circulating levels of endostatin in patients with pancreas cancer. *Int J Cancer* 122(12)**,** 2805-2810. doi: 10.1002/ijc.23468.

Olsen, R.S., Dimberg, J., Geffers, R., and Wågsäter, D. (2019). Possible Role and Therapeutic Target of PDGF-D Signalling in Colorectal Cancer. *Cancer Invest* 37(2)**,** 99-112. doi: 10.1080/07357907.2019.1576191.

Palumbo, J.S., Talmage, K.E., Massari, J.V., La Jeunesse, C.M., Flick, M.J., Kombrinck, K.W., et al. (2005). Platelets and fibrin(ogen) increase metastatic potential by impeding natural killer cell-mediated elimination of tumor cells. *Blood* 105(1)**,** 178-185. doi: 10.1182/blood-2004-06-2272.

Pang, B., Xu, X., Lu, Y., Jin, H., Yang, R., Jiang, C., et al. (2019). Prediction of new targets and mechanisms for quercetin in the treatment of pancreatic cancer, colon cancer, and rectal cancer. *Food Funct* 10(9)**,** 5339-5349. doi: 10.1039/c9fo01168d.

Parizadeh, S.M., Jafarzadeh-Esfehani, R., Fazilat-Panah, D., Hassanian, S.M., Shahidsales, S., Khazaei, M., et al. (2019). The potential therapeutic and prognostic impacts of the c-MET/HGF signaling pathway in colorectal cancer. *IUBMB Life* 71(7)**,** 802-811. doi: 10.1002/iub.2063.

Pasanisi, P., Venturelli, E., Morelli, D., Fontana, L., Secreto, G., and Berrino, F. (2008). Serum insulin-like growth factor-I and platelet-derived growth factor as biomarkers of breast cancer prognosis. *Cancer Epidemiol Biomarkers Prev* 17(7)**,** 1719-1722. doi: 10.1158/1055-9965.Epi-07-0654.

Pascual, G., Avgustinova, A., Mejetta, S., Martín, M., Castellanos, A., Attolini, C.S., et al. (2017). Targeting metastasis-initiating cells through the fatty acid receptor CD36. *Nature* 541(7635)**,** 41-45. doi: 10.1038/nature20791.

Peterson, J.E., Zurakowski, D., Italiano, J.E., Jr., Michel, L.V., Connors, S., Oenick, M., et al. (2012). VEGF, PF4 and PDGF are elevated in platelets of colorectal cancer patients. *Angiogenesis* 15(2)**,** 265-273. doi: 10.1007/s10456-012-9259-z.

Pilatova, K., Greplova, K., Demlova, R., Bencsikova, B., Klement, G.L., and Zdrazilova-Dubska, L. (2013). Role of platelet chemokines, PF-4 and CTAP-III, in cancer biology. *J Hematol Oncol* 6**,** 42. doi: 10.1186/1756-8722-6-42.

Pothula, S.P., Xu, Z., Goldstein, D., Pirola, R.C., Wilson, J.S., and Apte, M.V. (2020). Targeting HGF/c-MET Axis in Pancreatic Cancer. *Int J Mol Sci* 21(23). doi: 10.3390/ijms21239170.

Prokopchuk, O., Grünwald, B., Nitsche, U., Jäger, C., Prokopchuk, O.L., Schubert, E.C., et al. (2018). Elevated systemic levels of the matrix metalloproteinase inhibitor TIMP-1 correlate with clinical markers of cachexia in patients with chronic pancreatitis and pancreatic cancer. *BMC Cancer* 18(1)**,** 128. doi: 10.1186/s12885-018-4055-9.

Ren, J., He, J., Zhang, H., Xia, Y., Hu, Z., Loughran, P., et al. (2021). Platelet TLR4-ERK5 Axis Facilitates NET-Mediated Capturing of Circulating Tumor Cells and Distant Metastasis after Surgical Stress. *Cancer Res* 81(9)**,** 2373-2385. doi: 10.1158/0008-5472.Can-20-3222.

Riedl, J., Preusser, M., Nazari, P.M., Posch, F., Panzer, S., Marosi, C., et al. (2017). Podoplanin expression in primary brain tumors induces platelet aggregation and increases risk of venous thromboembolism. *Blood* 129(13)**,** 1831-1839. doi: 10.1182/blood-2016-06-720714.

Saito, M., Ichikawa, J., Ando, T., Schoenecker, J.G., Ohba, T., Koyama, K., et al. (2018). Platelet-Derived TGF-β Induces Tissue Factor Expression via the Smad3 Pathway in Osteosarcoma Cells. *J Bone Miner Res* 33(11)**,** 2048-2058. doi: 10.1002/jbmr.3537.

Sarrouilhe, D., and Mesnil, M. (2019). Serotonin and human cancer: A critical view. *Biochimie* 161**,** 46-50. doi: 10.1016/j.biochi.2018.06.016.

Sawai, H., Takeyama, H., Yamamoto, M., Furuta, A., Funahashi, H., Okada, Y., et al. (2003). Enhancement of integrins by interleukin-1alpha, and their relationship with metastatic and invasive behavior of human pancreatic ductal adenocarcinoma cells. *J Surg Oncol* 82(1)**,** 51-56. doi: 10.1002/jso.10187.

Shirai, T., Inoue, O., Tamura, S., Tsukiji, N., Sasaki, T., Endo, H., et al. (2017). C-type lectin-like receptor 2 promotes hematogenous tumor metastasis and prothrombotic state in tumor-bearing mice. *J Thromb Haemost* 15(3)**,** 513-525. doi: 10.1111/jth.13604.

Sierko, E., and Wojtukiewicz, M.Z. (2004). Platelets and angiogenesis in malignancy. *Semin Thromb Hemost* 30(1)**,** 95-108. doi: 10.1055/s-2004-822974.

Sleightholm, R.L., Neilsen, B.K., Li, J., Steele, M.M., Singh, R.K., Hollingsworth, M.A., et al. (2017). Emerging roles of the CXCL12/CXCR4 axis in pancreatic cancer progression and therapy. *Pharmacol Ther* 179**,** 158-170. doi: 10.1016/j.pharmthera.2017.05.012.

Stec, M., Baj-Krzyworzeka, M., Baran, J., Węglarczyk, K., Zembala, M., Barbasz, J., et al. (2015). Isolation and characterization of circulating micro(nano)vesicles in the plasma of colorectal cancer patients and their interactions with tumor cells. *Oncol Rep* 34(5)**,** 2768-2775. doi: 10.3892/or.2015.4228.

Takagi, S., Tsukamoto, S., Park, J., Johnson, K.E., Kawano, Y., Moschetta, M., et al. (2018). Platelets Enhance Multiple Myeloma Progression via IL-1β Upregulation. *Clin Cancer Res* 24(10)**,** 2430-2439. doi: 10.1158/1078-0432.Ccr-17-2003.

Tang, M., Jiang, L., Lin, Y., Wu, X., Wang, K., He, Q., et al. (2017). Platelet microparticle-mediated transfer of miR-939 to epithelial ovarian cancer cells promotes epithelial to mesenchymal transition. *Oncotarget* 8(57)**,** 97464-97475. doi: 10.18632/oncotarget.22136.

Toth, B., Liebhardt, S., Steinig, K., Ditsch, N., Rank, A., Bauerfeind, I., et al. (2008). Platelet-derived microparticles and coagulation activation in breast cancer patients. *Thromb Haemost* 100(4)**,** 663-669.

Trajkovic-Arsic, M., Kalideris, E., and Siveke, J.T. (2013). The role of insulin and IGF system in pancreatic cancer. *J Mol Endocrinol* 50(3)**,** R67-74. doi: 10.1530/jme-12-0259.

van der Zee, P.M., Biró, E., Ko, Y., de Winter, R.J., Hack, C.E., Sturk, A., et al. (2006). P-selectin- and CD63-exposing platelet microparticles reflect platelet activation in peripheral arterial disease and myocardial infarction. *Clin Chem* 52(4)**,** 657-664. doi: 10.1373/clinchem.2005.057414.

Visentin, B., Vekich, J.A., Sibbald, B.J., Cavalli, A.L., Moreno, K.M., Matteo, R.G., et al. (2006). Validation of an anti-sphingosine-1-phosphate antibody as a potential therapeutic in reducing growth, invasion, and angiogenesis in multiple tumor lineages. *Cancer Cell* 9(3)**,** 225-238. doi: 10.1016/j.ccr.2006.02.023.

Wang, C.S., Wu, T.L., Tsao, K.C., and Sun, C.F. (2006). Serum TIMP-1 in gastric cancer patients: a potential prognostic biomarker. *Ann Clin Lab Sci* 36(1)**,** 23-30.

Wang, Y., Sun, Y., Li, D., Zhang, L., Wang, K., Zuo, Y., et al. (2013). Platelet P2Y12 is involved in murine pulmonary metastasis. *PLoS One* 8(11)**,** e80780. doi: 10.1371/journal.pone.0080780.

Westphalen, C.B., and Olive, K.P. (2012). Genetically engineered mouse models of pancreatic cancer. *Cancer J* 18(6)**,** 502-510. doi: 10.1097/PPO.0b013e31827ab4c4.

Xiao, G.G. (2020). Targeting Serotonin System in Pancreatic Cancer. *Pancreas* 49(1)**,** e1. doi: 10.1097/mpa.0000000000001417.

Yenigürbüz, F.D., Kızmazoğlu, D., Ateş, H., Erdem, M., Tüfekçi, Ö., Yılmaz, Ş., et al. (2019). Analysis of apoptotic, platelet-derived, endothelial-derived, and tissue factor-positive microparticles of children with acute lymphoblastic leukemia during induction therapy. *Blood Coagul Fibrinolysis* 30(4)**,** 149-155. doi: 10.1097/mbc.0000000000000811.

Yu, L.X., Yan, L., Yang, W., Wu, F.Q., Ling, Y., Chen, S.Z., et al. (2014). Platelets promote tumour metastasis via interaction between TLR4 and tumour cell-released high-mobility group box1 protein. *Nat Commun* 5**,** 5256. doi: 10.1038/ncomms6256.

Yu, M., Li, T., Li, B., Liu, Y., Wang, L., Zhang, J., et al. (2020). Phosphatidylserine-exposing blood cells, microparticles and neutrophil extracellular traps increase procoagulant activity in patients with pancreatic cancer. *Thromb Res* 188**,** 5-16. doi: 10.1016/j.thromres.2020.01.025.

Zhang, H., Jiang, P., Zhang, C., Lee, S., Wang, W., and Zou, H. (2018). PAR4 overexpression promotes colorectal cancer cell proliferation and migration. *Oncol Lett* 16(5)**,** 5745-5752. doi: 10.3892/ol.2018.9407.
